# Impact of Immediate vs Delayed Dental Implants on Survival, Patient Satisfaction, and Quality of Life

**DOI:** 10.3290/j.ohpd.c_2438

**Published:** 2026-02-10

**Authors:** Yi Yang, Shuncheng Zhou, Yihui Ma, Xiang Wang, Jinfang Chen, Qingshan Dong

**Affiliations:** a Yi Yang Dentist, Department of Stomatology, General Hospital of Central Theater Command, Wuhan 430012, Hubei Province, China. Conception and design, or analysis and interpretation of the data.; b Shuncheng Zhou Dentist, Department of Stomatology, General Hospital of Central Theater Command, Wuhan 430012, Hubei Province, China. Drafting the paper, revising it critically for intellectual content.; c Yihui Ma Dentist, Department of Stomatology, General Hospital of Central Theater Command, Wuhan 430012, Hubei Province, China. Drafting the paper, revising it critically for intellectual content.; d Xiang Wang Dentist, Department of Stomatology, General Hospital of Central Theater Command, Wuhan 430012, Hubei Province, China. Final approval of the version to be published..; e Jinfang Chen Dentist, Department of Stomatology, General Hospital of Central Theater Command, Wuhan 430012, Hubei Province, China. Final approval of the version to be published.; f Qingshan Dong Dentist, Department of Stomatology, General Hospital of Central Theater Command, Wuhan 430012, Hubei Province, China. Conception and design, or analysis and interpretation of the data.

**Keywords:** delayed implantation, evaluation of aesthetic results, immediate implantation, implant survival, patient satisfaction, quality of life

## Abstract

**Purpose:**

This randomised controlled trial investigated whether immediate placement confers any advantage over delayed placement with respect to implant survival (primary outcome), peri-implant health, patient-reported satisfaction (VAS) and oral-health-related quality of life (OHIP-14).

**Methods and Materials:**

This study included 220 patients with missing teeth, randomly divided into a control group (n = 110) and an observation group (n = 110) using a random number table (block = 10, concealed allocation). Baseline variables (age, sex, BMI, tooth site, aetiology and oral hygiene habits) showed no statistically significant between-group differences (all P > 0.05). The control group received delayed implants, while the observation group received immediate implants. Outcomes measured included implant survival rate, periodontal indexes (PD, mPLI, mSBI), aesthetic evaluation (PES, WES), quality of life (OHIP-14), and patient satisfaction (VAS). Attachment level in the satisfaction assessment refers to the perceived gingival margin position relative to the restoration, evaluated subjectively by patients using a visual analogue scale rather than clinical probing measurements.

**Results:**

At 1 year, implant survival rates were 95.45% (105/110) in the immediate group versus 92.73% (102/110) in the delayed group, with no statistically significant difference (χ^2^ = 0.736, P = 0.391). The observation group demonstrated significantly lower mean values compared to the control group for PD (3.16 ± 0.51 mm vs 3.39 ± 0.59 mm), mPLI (0.95 ± 0.38 vs 1.21 ± 0.54), and mSBI (0.85 ± 0.47 vs 1.01 ± 0.35) (all P < 0.05). The PES total scores were significantly higher in the observation group than the control group (12.18 ± 1.13 vs 11.34 ± 1.30, P < 0.001), as were WES total scores (7.78 ± 0.99 vs 7.23 ± 1.10, P < 0.001). After 1 year of implantation, OHIP-14 total scores in the observation group were significantly lower than those in the control group (3.20 ± 1.33 vs 4.15 ± 1.23, P < 0.001). The satisfaction scores for Attachment Level (8.12 ± 1.05 vs 7.45 ± 1.18), Colour (8.67 ± 0.89 vs 8.15 ± 1.12), and Chewing Function (8.89 ± 0.94 vs 8.23 ± 1.08) were significantly higher in the observation group than in the control group (all P < 0.05).

**Conclusion:**

Based on the primary outcome of 1-year implant survival, there was no significant difference between immediate and delayed implantation. However, immediate implantation demonstrated superior secondary outcomes, including periodontal-related indexes, aesthetic effects, quality of life, and patient satisfaction. These findings suggest that immediate implantation may offer clinical advantages in early functional and aesthetic outcomes without compromising implant survival, providing patients with reduced treatment duration and improved peri-implant tissue preservation.

In the field of dentistry, missing teeth is a common condition that seriously affects patients’ masticatory function, aesthetics, and quality of life.^35,20^ Among adults, the proportion of missing teeth due to various types of oral diseases and trauma is on the rise, which not only brings considerable inconvenience to patients’ daily diet, pronunciation and other physiological functions, but also negatively affects their psychological health and social interactions.^22,35^ Dental implant technology, as a reliable means of restoration, has made significant progress in recent years, bringing patients a solution that is close to the function and aesthetics of natural teeth.^13^

According to the classification proposed by Hämmerle and colleagues, immediate dental implants refer to implant placement at the time of tooth extraction, while delayed dental implants involve implant placement after complete healing of the extraction socket, typically 3 months or more post-extraction.^9^ Delayed dental implants give sufficient healing time to the extraction wound to ensure complete healing of soft and hard tissues for successful osseointegration.^9^ However, studies have shown that the need for complete post-extraction healing prior to implant placement is not essential,^3,40^ and furthermore, while waiting for the socket to heal, physiologic resorption of the alveolar bone may occur, which affects the long-term stability of the implant, and the patient will have to tolerate aesthetic and chewing limitations during the period of missing teeth.^41^ In contrast, immediate dental implantation has the advantages of reducing the number of patient visits and shortening the treatment period by virtue of its ability to place the implant immediately after tooth extraction, and it can preserve the original structure and blood flow of the alveolar bone to a certain extent, which is theoretically favourable for the early integration of the implant with the alveolar bone.^37,43^ Immediate dental implants have been reported to minimise post-extraction bone resorption, thereby preserving periodontal structure and bringing better aesthetic treatment results.^7,8,46^ Despite the fact that these two implant modalities have been used in clinical practice for a long time, there is still controversy about their effects on implant survival, patient satisfaction, and quality of life.

Recent systematic reviews and meta-analyses have provided important insights into implant placement timing.^19,30^ Long-term outcomes of post-extraction alveolar ridge preservation followed by delayed implant placement have demonstrated favourable results, though the need for multiple surgical interventions remains a consideration.1 Studies on peri-implant conditions have emphasised the importance of diagnostic methods and parameters for monitoring long-term implant health.^23^ The radiographic assessment of peri-implant sites has become increasingly sophisticated, allowing for better evaluation of bone preservation strategies.^38^ Furthermore, research on tooth- and implant-related prognostic factors has highlighted the significance of treatment timing in achieving optimal outcomes.^28^ The European Federation of Periodontology has contributed substantially to advancing our understanding of implant treatment protocols and their long-term implications.^39^ Recent evidence suggests that bone-level implants restored with appropriate abutments may demonstrate reduced marginal bone loss, which is particularly relevant when considering immediate versus delayed placement strategies.^44^ Additionally, the long-term surgical treatment outcomes of peri-implantitis underscore the importance of proper initial implant placement and tissue management.^24^

In view of the advantages and disadvantages of the two implant modalities, there have been insufficient comparative studies on implant survival, periodontal health maintenance, aesthetic results, patient satisfaction, and improvement in quality of life. The null hypothesis of this study was that there would be no significant difference in 1-year implant survival rate between immediate and delayed dental implantation. The aim of this randomised controlled trial was to investigate whether immediate versus delayed dental implantation differs in terms of implant survival rate at 1 year (primary outcome), as well as secondary outcomes including peri-implant health, aesthetic results, patient satisfaction, and quality of life, thereby providing clinicians with a more scientific and comprehensive basis for choosing dental implantation treatment plans and achieving the goal of providing patients with optimal treatment results.

## MATERIALS AND METHODS

### Study Design and Population

This was a parallel-group randomised controlled trial with an allocation ratio of 1:1. Between January 2022 and October 2023, 256 patients with missing teeth were prospectively screened at our hospital. Following application of inclusion and exclusion criteria, 232 patients were eligible, of whom 12 refused to participate and were excluded. The remaining 220 patients with missing teeth were enrolled as study subjects and randomly allocated to the control group (n = 110, delayed implant protocol) or the observation group (n = 110, immediate implant protocol) according to a random number table method.

### Ethical Considerations

The study was reviewed and approved by the Ethics Committee of our hospital (Approval Number: GN-CTC-121) in accordance with the Declaration of Helsinki. All participants provided written informed consent prior to enrollment. The consent process involved a detailed explanation of the study’s purpose, procedures, potential risks, benefits, and alternatives in accessible language. Vulnerable populations (pregnant women, prisoners, and cognitively impaired individuals) were excluded from participation. Data were de-identified using unique study codes and stored on encrypted servers under HIPAA/GDPR requirements. A Data and Safety Monitoring Board reviewed adverse events quarterly. Participants received a travel stipend (USD 15) deemed non-coercive. No conflicts of interest were present that could influence study conduct or reporting. The study complied with 45 CFR 46 and ICH-GCP guidelines.

### Inclusion and Exclusion Criteria

Inclusion criteria were as follows: patients aged ≥ 18 years; only a single missing tooth; normal occlusal relationship without parafunctional habits such as bruxism; good alveolar bone quality at the missing site as assessed by cone-beam computed tomography showing adequate bone density ( > 500 HU) and sufficient bone volume (minimum 10 mm height and 6 mm width); good compliance, able to follow-up as required; good general health; and non-smokers or light smokers consuming fewer than 10 cigarettes per day with no history of periodontitis.

Exclusion criteria were as follows: serious systemic or bone metabolic diseases, such as uncontrolled heart disease, hypertension, or osteoporosis; parafunctional oral habits such as severe bruxism or clenching; patients who are allergic to the medications or materials used; patients with mental illness or serious heart, liver, kidney and other organ complications and insufficiencies; existing malignant tumours; pregnant or lactating women; patients with uncontrolled chronic diseases such as heart disease, hypertension, or diabetes; and patients with incomplete medical records.

### Randomisation and Allocation Concealment

Randomisation was implemented by an independent researcher not involved in patient recruitment or treatment using permuted blocks of size 10. Sealed opaque envelopes containing group assignments were prepared and sequentially numbered. After obtaining informed consent and confirming eligibility, the envelope was opened by the independent researcher to reveal group allocation. *A priori* power analysis (α = 0.05, β = 0.20, two-sided) indicated that 98 participants per arm would detect a 10-point OHIP-14 difference with adequate power; 110 per arm were enrolled to compensate for anticipated 10% attrition. Based on pilot data, a 10-point difference in OHIP-14 was considered clinically meaningful.

### Treatment

Before implantation, patients in both groups underwent comprehensive oral health examination, imaging examination (panoramic radiography and cone-beam computed tomography), and basic oral treatment including professional cleaning and treatment of any active caries or periodontal disease.

Patients in the control group underwent delayed implantation. The procedure was as follows: first, the affected tooth was extracted using a minimally invasive technique that separated the gingiva to ensure complete removal of the root, and the socket was thoroughly curetted to remove residual granulation tissue and periodontal ligament remnants, followed by hemostasis by compression to ensure that there was no active bleeding in the socket. The patient waited for 3 months after extraction, during which time the alveolar bone gradually healed in preparation for subsequent implant placement. At the follow-up visit, an incision was made at the apex of the proximal and distal mesial alveolar ridge and flaps were reflected and precisely positioned using a pilot drill, followed by step-by-step preparation of the implant site using sequential drills. Bone-level implants (Straumann Bone Level Tapered, Institut Straumann, Basel, Switzerland) were used with the implant shoulder positioned 3 mm below the ideal gingival margin and at least 1.5 mm away from the adjacent natural tooth, leaving a gap of 2 mm between the buccal aspect of the implant and the bone wall; if the gap was greater than 2 mm, the site was filled with deproteinised bovine bone mineral (Bio-Oss, Geistlich Pharma, Wolhusen, Switzerland) to ensure adequate support and stability. The implant was placed, ensuring that the insertion torque was ≥35 N·cm to ensure initial stability. Finally, the mucosa was repositioned using careful flap management to minimise damage to the surrounding tissues, and the wound was closed with tension-free 4-0 polyglycolic acid sutures to promote healing and avoid infection and other complications. Postoperative instructions included a soft diet for 2 weeks, oral hygiene with chlorhexidine 0.12% rinse twice daily for 2 weeks, and analgesics (ibuprofen 400 mg as needed). Sutures were removed at 10–14 days. Patients were monitored at 1 week, 2 weeks, 1 month, 3 months, 6 months, and 12 months post-surgery.

Patients in the observation group underwent immediate implantation. The procedure was as follows: under local anaesthesia, teeth were extracted atraumatically and granulation tissue in the extraction sockets was cleared and rinsed with 0.9% sodium chloride solution. Bone-level implants (Straumann Bone Level Tapered) were placed in a restoratively oriented manner, with their positions, directions and specifications determined according to pre-operative planning. The palatal bone wall of the extraction socket was used as the primary stabilisation site, and implants were placed after step-by-step site preparation, with the implant apex positioned 3.0–5.0 mm beyond the apical extent of the alveolar socket; the implant platform was positioned 0.5 mm below the alveolar crest; at least 1.0 mm of bone was maintained on the buccal and lingual aspects; and insertion torque was ≥35 N·cm. Gaps between the implant and socket walls were filled with deproteinised bovine bone mineral (Bio-Oss) and covered with a resorbable collagen membrane (Bio-Gide, Geistlich Pharma). The mucoperiosteal flap was released on the buccal side to achieve tension-free closure, advanced coronally, and sutured with 4-0 polyglycolic acid sutures to ensure complete coverage of the membrane and bone graft. Postoperative care, medications, and follow-up schedule were identical to the delayed group.

### Baseline Data Collection

Data on patients’ age, gender, body mass index (BMI), location of missing teeth (premolar region, posterior region), cause of tooth loss (caries, trauma, or chronic apical periodontitis), and oral hygiene habits (brushing two or more times a day, use of dental floss) were collected at baseline.

### Outcome Measures

The primary outcome was the implant survival rate at 1 year after functional loading. The combination of the implant and the alveolar bone was observed by periapical radiographs to determine whether the implant survived. The criteria for implant survival, consistent with established consensus guidelines, were: no clinical mobility of the implant, no peri-implant radiolucency on radiographs, and evidence of osseointegration between the implant and alveolar bone.^17^

Secondary outcomes included periodontal parameters, aesthetic scores, quality-of-life measures, and patient satisfaction, all assessed according to the Implant Dentistry Core Outcome Set and Measurements (ID-COSM) international consensus report.^45^ Probing depth (PD), modified plaque index (mPLI), and modified sulcus bleeding index (mSBI) were measured at 1 year after restoration in both groups. A periodontal probe was used to measure the PD of the patients, and the PD values at four sites around each implant (mesial, distal, buccal, lingual) were recorded and the mean value was calculated. The mPLI was used to assess the patients’ peri-implant plaque accumulation, with scores ranging from 0 (no plaque) to 3 (abundant soft deposits in the gingival sulcus). The mSBI was used to assess peri-implant gingival bleeding, with scores ranging from 0 (healthy gingiva with no bleeding) to 3 (severe gingival inflammation with profuse bleeding).

The pink aesthetic score (PES) and white aesthetic score (WES) were used to evaluate the aesthetic effect after treatment.^5,25^ The PES evaluated the aesthetic effect of the peri-implant soft tissues, including seven indicators: proximal mesial papilla height, distal mesial papilla height, labial mucosal curvature, labial mucosal height, labial root protrusion, soft tissue colour, and soft tissue texture. WES includes five indicators of the size, shape, colour, surface texture and transparency of the restorations, with a total score of 10 points using a 0–2 point grading system, and the higher the score, the better the aesthetic effect of the restorations. All patients were scored by the same calibrated examiner who was blinded to group allocation.

The quality of life of patients was assessed using the Chinese version of the Oral Health Impact Profile-14 (OHIP-14).^47,48^ The Chinese version of the OHIP-14 has been demonstrated to have strong reliability (Cronbach’s alpha = 0.93) and validity (corrected item-total correlation varied from 0.53 to 0.71). In the present sample, Cronbach’s α = 0.94 and test-retest ICC = 0.91 (n = 30, 2-week interval), confirming internal consistency and stability. The scale consists of 14 items in seven dimensions: functional limitations, physical pain, psychological discomfort, physical disability, psychological disability, social disability, and disability. Each item was scored on a 5-point Likert scale: never (0), rarely (1), occasionally (2), fairly often (3) and often (4). Higher scores indicate a more negatively affected quality of life. The OHIP-14 scale was assessed at baseline before treatment and at 1 year after implant restoration to compare the changes in quality of life between the two groups.

Patient satisfaction evaluation was conducted using the visual analogue scale (VAS) to assess the subjective satisfaction of patients at 1 year after implant restoration, including four domains: attachment level (referring to the perceived gingival margin position and overall soft tissue integration with the restoration as subjectively evaluated by patients), colour match with adjacent teeth, overall aesthetics, and masticatory function. Each item was scored from 0 (completely dissatisfied) to 10 (completely satisfied), with higher scores indicating greater patient satisfaction.

### Statistical Analysis

Data were statistically analysed and graphed using GraphPad Prism 9.5.0 software (GraphPad Software, San Diego, CA, USA). The Kolmogorov-Smirnov test was used to test for normal distribution. For normally distributed continuous variables, the independent sample t-test was used for between-group comparisons, and the paired t-test was used for within-group comparisons before and after treatment. For non-normally distributed data, the Wilcoxon Signed Rank Test was used for within-group comparisons, and the Mann–Whitney U test was used for between-group comparisons. Categorical data were expressed as frequencies and percentages, and comparisons between groups were made using the chi-square test or Fisher’s exact test as appropriate. P-values were two-sided, and differences were considered statistically significant at P < 0.05. Additionally, multivariate logistic regression tested the independent effect of implant timing on 1-year survival after adjusting for age, sex and tooth region; linear regression adjusted OHIP-14 and VAS outcomes for the same covariates. Results are reported as mean ± standard deviation for continuous variables unless otherwise specified.

### Data Availability

De-identified individual-participant data (IPD) and analytic code will be made available on reasonable request to the corresponding author, 6 months after publication, in compliance with HIPAA/GDPR regulations.

## RESULTS

### Comparison of Baseline Data Between the Two Groups of Patients

Baseline characteristics were comparable between groups (Table 1). Two hundred and twenty patients with missing teeth were finally included in this study, and the patients were divided into a control group (n = 110) and an observation group (n = 110) according to the randomised number table method. The delayed implant programme was used in the control group, and the immediate implant programme was used in the observation group. There was no significant difference between the two groups in terms of age (40.46 ± 6.33 years vs 41.65 ± 5.91 years), gender distribution (56.36% male vs 52.73% male), BMI (22.59 ± 1.90 kg/m^2^ vs 22.70 ± 2.23 kg/m^2^), location of affected teeth, cause of tooth loss, and oral hygiene habits (all P > 0.05), as shown in Table 1.

**Table 1:** Comparison of baseline data between the two groups of patients

Parameter	Control group (n = 110)	Observation group (n = 110)	P
Age (years)	40.46 ± 6.33	41.65 ± 5.91	0.154
Gender (n,%)			
M	62 (56.36%)	58 (52.73%)	0.588
F	48 (43.64%)	52 (47.27%)	
BMI (kg/m²)	22.59 ± 1.90	22.70 ± 2.23	0.687
Position of the affected tooth (n,%)			
premolar region	40 (36.36%)	44 (40.00%)	0.579
posterior region	70 (63.64%)	66 (60.00%)	
Cause of missing teeth (n,%)			
dental caries	66 (60.00%)	61 (55.45%)	0.784
trauma	24 (21.82%)	26 (23.64%)	
Chronic periapical inflammation	20 (18.18%)	23 (20.91%)	
Oral hygiene practices (cases, %)			
Brush teeth 2 or more times a day	83 (75.45%)	79 (71.82%)	0.541
Use dental floss	44 (40.00%)	39 (35.45%)	0.487
Note: BMI: Body Mass Index. P < 0.05 was taken as a statistically significant difference.

### Comparison of Implant Survival Rate Between the Two Groups

One year after the completion of implantation, 102 cases of implant restorations in the control group were successful and 8 cases failed, yielding an implant survival rate of 92.73% (95% CI: 86.2-96.8%); 105 cases of implant restorations in the observation group were successful and 5 cases failed, yielding an implant survival rate of 95.45% (95% CI: 89.7–98.5%). The difference between the two groups in terms of implant survival rate at the end of 1 year was not statistically significant (χ^2^ = 0.736, P = 0.391). Multivariate logistic regression adjusting for age, sex, and tooth region confirmed that implant timing (immediate vs delayed) was not independently associated with 1-year survival (adjusted OR = 1.82, 95% CI: 0.56–5.91, P = 0.321).

### Comparison of Periodontal-Related Indexes Before and After Restoration in Two Groups of Patients

**Fig 1:**
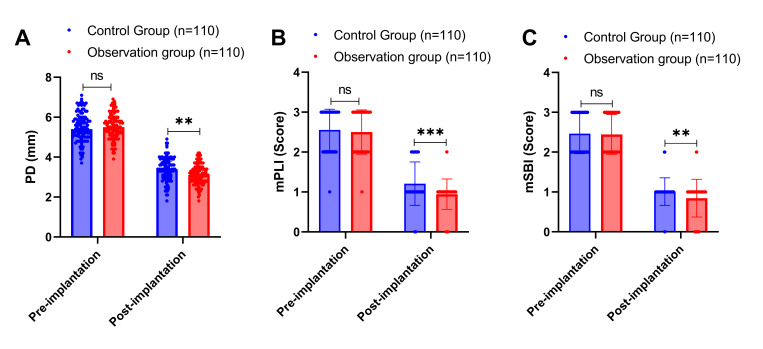
Comparison of periodontal-related indexes before and after restoration in the two groups of patients. Note: PD: Probing Depth; mPLI: Modified Plaque Index; mSBI: Modified Sulcus Bleeding Index. Measures with normal distribution were analysed before and after treatment by paired t-test, and between groups by independent samples t-test; measures without normal distribution were analysed before and after treatment by Wilcoxon Signed Rank Test, and between groups by Mann–Whitney U test. ns indicates P > 0.05, ** indicates P < 0.01, and *** indicates P < 0.001.

We compared the probing depth (PD), modified plaque index (mPLI), and modified sulcus bleeding index (mSBI) before and after implantation in both groups. Before implantation, there was no statistically significant difference in the baseline levels of PD, mPLI, and mSBI between the two groups (P > 0.05) (Fig 1). At 1 year after implantation, the mean value of PD in the control group was 3.39 ± 0.59 mm, the mean value of mPLI was 1.21 ± 0.54, and the mean value of mSBI was 1.01 ± 0.35, representing mean changes from baseline of –0.48 mm, –0.63, and –0.52, respectively. In the observation group, the mean value of PD was 3.16 ± 0.51 mm, the mean value of mPLI was 0.95 ± 0.38, and the mean value of mSBI was 0.85 ± 0.47, representing mean changes from baseline of –0.71 mm, –0.89, and –0.68, respectively. These results showed that the levels of PD, mPLI, and mSBI in both groups were significantly lower than those before implantation (all P < 0.001), and importantly, the observation group demonstrated significantly greater improvements with lower absolute values of PD (mean difference: –0.23 mm, 95% CI: –0.39 to –0.07, P = 0.005), mPLI (mean difference: –0.26, 95% CI: –0.40 to –0.12, P < 0.001), and mSBI (mean difference: –0.16, 95% CI: –0.27 to –0.05, P = 0.004) compared to the control group.

### Comparison of Aesthetic Scores Before and After Restoration in Two Groups of Patients

Before implantation, the differences in the PES scores (proximal mesial gingival papilla height, distal mesial gingival papilla height, labial mucosal curvature, labial mucosal height, labial root surface protrusion, soft tissue colour, and soft tissue texture) and total scores of the patients in the two groups were not statistically significant when compared between groups (P > 0.05). After 1 year of implantation, all individual PES scores and total scores of patients in both groups were significantly higher than those before implantation (P < 0.001). Notably, the observation group demonstrated significantly higher scores than the control group in labial lateral mucosal curvature (mean difference: +0.17, 95% CI: 0.03 to 0.31, P = 0.020), labial lateral mucosal height (mean difference: +0.24, 95% CI: 0.09 to 0.39, P = 0.002), soft tissue colour (mean difference: +0.18, 95% CI: 0.08 to 0.28, P < 0.001), and soft tissue texture (mean difference: +0.21, 95% CI: 0.11 to 0.31, P < 0.001). The total PES score in the observation group was significantly higher than in the control group (12.18 ± 1.13 vs 11.34 ± 1.30, mean difference: +0.84, 95% CI: 0.51 to 1.17, P < 0.001), as shown in Table 2.

**Table 2:** Comparison of PES scores before and after implantation in the two groups of patients

Parameter	Timing	Control group (n = 110)	Observation group (n = 110)	Mean change control	Mean change observation	P
Proximal mesial gingival papilla height	Pre-implantation	1.00 ± 0.19	1.04 ± 0.30			0.281
	Post-implantation	1.58 ± 0.50	1.66 ± 0.47	+0.58	+0.62	0.212
	P	< 0.001	< 0.001			
Distal mesial gingival papilla height	Pre-implantation	1.17 ± 0.50	1.25 ± 0.58			0.267
	Post-implantation	1.67 ± 0.47	1.66 ± 0.50	+0.50	+0.41	0.743
	P	< 0.001	< 0.001			
Labial lateral mucosal curvature	Pre-implantation	1.16 ± 0.53	1.17 ± 0.56			0.776
	Post-implantation	1.57 ± 0.55	1.74 ± 0.46	+0.41	+0.57	0.020
	P	< 0.001	< 0.001			
Labial lateral mucosa height	Pre-implantation	1.09 ± 0.42	1.11 ± 0.49			0.725
	Post-implantation	1.44 ± 0.60	1.68 ± 0.49	+0.35	+0.57	0.002
	P	< 0.001	< 0.001			
Labial root surface protrusion	Pre-implantation	1.05 ± 0.34	1.03 ± 0.37			0.713
	Post-implantation	1.71 ± 0.46	1.68 ± 0.47	+0.66	+0.65	0.662
	P	< 0.001	< 0.001			
Soft tissue colour	Pre-implantation	1.12 ± 0.35	1.06 ± 0.37			0.275
	Post-implantation	1.71 ± 0.46	1.89 ± 0.31	+0.59	+0.83	< 0.001
	P	< 0.001	< 0.001			
Soft tissue texture	Pre-implantation	1.26 ± 0.48	1.20 ± 0.48			0.424
	Post-implantation	1.66 ± 0.48	1.87 ± 0.33	+0.40	+0.67	< 0.001
	P	< 0.001	< 0.001			
PES total score	Pre-implantation	7.84 ± 1.12	7.86 ± 1.20			0.759
	Post-implantation	11.34 ± 1.30	12.18 ± 1.13	+3.50	+4.32	< 0.001
	P	< 0.001	< 0.001			
Note: PES: Pink esthetic score. Differences were considered statistically significant at P < 0.05.

Before implantation, there was no statistically significant difference between the WES scores (size, shape, colour, surface texture and transparency of the restorations) and the total scores of the two groups (P > 0.05). At 1 year after implantation, the observation group demonstrated significantly higher scores than the control group in restoration shape (mean difference: +0.35, 95% CI: 0.24 to 0.46, P < 0.001) and surface texture (mean difference: +0.15, 95% CI: 0.03 to 0.27, P = 0.012). The total WES score in the observation group was significantly higher than in the control group (7.78 ± 0.99 vs 7.23 ± 1.10, mean difference: +0.55, 95% CI: 0.28 to 0.82, P < 0.001), as shown in Table 3.

**Table 3:** Comparison of WES scores before and after implantation in the two groups of patients

Parameter	Timing	Control group (n = 110)	Observation group (n = 110)	Mean change control	Mean change observation	P
Restoration size	Pre-implantation	1.46 ± 0.50	1.53 ± 0.50			0.347
	Post-implantation	1.59 ± 0.49	1.58 ± 0.50	+0.13	+0.05	0.892
	P	0.103	0.497			
Restoration shape	Pre-implantation	1.14 ± 0.34	1.22 ± 0.46			0.117
	Post-implantation	1.51 ± 0.50	1.86 ± 0.35	+0.37	+0.64	< 0.001
	P	< 0.001	< 0.001			
Restoration colour	Pre-implantation	1.43 ± 0.50	1.46 ± 0.50			0.589
	Post-implantation	1.53 ± 0.50	1.56 ± 0.50	+0.10	+0.10	0.590
	P	0.193	0.161			
Restoration surface texture	Pre-implantation	1.07 ± 0.32	1.11 ± 0.34			0.423
	Post-implantation	1.22 ± 0.41	1.37 ± 0.49	+0.15	+0.26	0.012
	P	0.011	< 0.001			
Transparency of restorations	Pre-implantation	1.36 ± 0.48	1.34 ± 0.47			0.673
	Post-implantation	1.38 ± 0.49	1.41 ± 0.49	+0.02	+0.07	0.681
	P	0.890	0.302			
WES total score	Pre-implantation	6.46 ± 0.92	6.66 ± 1.06			0.239
	Post-implantation	7.23 ± 1.10	7.78 ± 0.99	+0.77	+1.12	< 0.001
	P	< 0.001	< 0.001			
Note: WES: White esthetic score. Differences were considered statistically significant at P < 0.05.

### Comparison of Quality of Life After Implantation in Two Groups of Patients

We used the Oral Health Impact Profile-14 (OHIP-14) to assess patients’ quality of life. Before implantation, there was no statistically significant difference between the OHIP-14 scores across all seven dimensions (functional limitations, physical pain, psychological discomfort, physical impairment, social impairment, psychological impairment, and disability) and total scores of the two groups (P > 0.05). At 1 year after implantation, all OHIP-14 dimensional scores and total scores in both groups were significantly lower than those of the pre-implantation period (P < 0.001), indicating improved quality of life. Comparing between groups at 1 year, the observation group demonstrated significantly lower (better) scores than the control group in functional limitations (mean difference: –0.18, 95% CI: –0.32 to –0.04, P = 0.010), physical pain (mean difference: –0.25, 95% CI: –0.39 to –0.11, P < 0.001), psychological discomfort (mean difference: –0.33, 95% CI: –0.48 to –0.18, P < 0.001), and psychological disorders (mean difference: –0.15, 95% CI: –0.27 to –0.03, P = 0.018). The total OHIP-14 score was significantly lower in the observation group than in the control group (3.20 ± 1.33 vs 4.15 ± 1.23, mean difference: –0.95, 95% CI: –1.30 to –0.60, P < 0.001), as shown in Table 4. Linear regression analysis adjusting for age, sex, and tooth region confirmed that immediate implantation was independently associated with lower (better) OHIP-14 total scores (adjusted β coefficient: –0.89, 95% CI: –1.26 to –0.52, P < 0.001).

**Table 4:** Comparison of quality of life before and after treatment between the two groups of patients

Parameter	Timing	Control group (n = 110)	Observation group (n = 110)	Mean change control	Mean change observation	P
Functional limitations (score)	Pre-implantation	1.30 ± 0.53	1.33 ± 0.54			0.694
	Post-implantation	0.64 ± 0.48	0.46 ± 0.50	–0.66	–0.87	0.010
	P	< 0.001	< 0.001			
Physical pain (score)	Pre-implantation	2.08 ± 0.91	2.16 ± 1.02			0.676
	Post-implantation	0.83 ± 0.43	0.58 ± 0.53	–1.25	–1.58	< 0.001
	P	< 0.001	< 0.001			
Psychological discomfort (score)	Pre-implantation	1.60 ± 0.68	1.55 ± 0.77			0.760
	Post-implantation	0.69 ± 0.57	0.36 ± 0.48	–0.91	–1.19	< 0.001
	P	< 0.001	< 0.001			
Physical disorders (score)	Pre-implantation	1.23 ± 0.62	1.29 ± 0.61			0.442
	Post-implantation	0.48 ± 0.52	0.45 ± 0.53	–0.75	–0.84	0.548
	P	< 0.001	< 0.001			
Socialisation disorder (score)	Pre-implantation	1.73 ± 0.59	1.66 ± 0.63			0.368
	Post-implantation	0.60 ± 0.51	0.64 ± 0.50	–1.13	–1.02	0.588
	P	< 0.001	< 0.001			
Mental disorders (score)	Pre-implantation	2.17 ± 1.00	2.26 ± 0.99			0.645
	Post-implantation	0.45 ± 0.50	0.30 ± 0.46	–1.72	–1.96	0.018
	P	< 0.001	< 0.001			
Disability (score)	Pre-implantation	1.14 ± 0.57	1.15 ± 0.56			0.916
	Post-implantation	0.45 ± 0.52	0.41 ± 0.51	–0.69	–0.74	0.502
	P	< 0.001	< 0.001			
OHIP-14 total (score)	Pre-implantation	11.25 ± 2.03	11.38 ± 2.11			0.539
	Post-implantation	4.15 ± 1.23	3.20 ± 1.33	–7.10	–8.18	< 0.001
	P	< 0.001	< 0.001			
Note: OHIP-14: Oral Health Impact Profile-14. Differences are considered statistically significant at P< 0.05.

### Comparison of Post-Implantation Satisfaction Between the Two Groups of Patients

**Fig 2:**
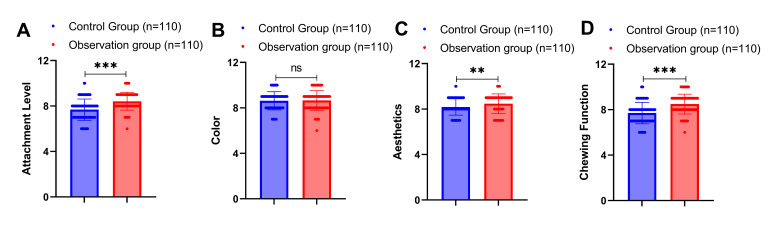

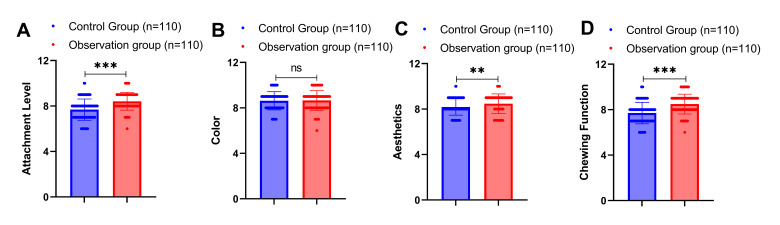
Comparison of post-implantation satisfaction between the two groups of patients. Note: Mann-Whitney U test between groups for non-normally distributed measures. ns indicates P > 0.05, ** indicates P < 0.01, and *** indicates P < 0.001.

One year after implantation, patient satisfaction was assessed across four domains using VAS. There was no statistically significant difference in aesthetic satisfaction scores between the two groups (8.34 ± 1.12 vs 8.11 ± 1.23, P = 0.142) (Fig 2). However, the observation group reported significantly higher satisfaction scores compared to the control group in attachment level (8.12 ± 1.05 vs 7.45 ± 1.18, mean difference: +0.67, 95% CI: 0.36 to 0.98, P < 0.001), colour match (8.67 ± 0.89 vs 8.15 ± 1.12, mean difference: +0.52, 95% CI: 0.24 to 0.80, P < 0.001), and chewing function (8.89 ± 0.94 vs 8.23 ± 1.08, mean difference: +0.66, 95% CI: 0.38 to 0.94, P < 0.001). Linear regression adjusting for covariates confirmed these associations remained significant.

### Representative Cases

#### Case 1 (control group – delayed implantation)

A 42-year-old female patient presented with a non-restorable mandibular first molar due to severe caries. Following atraumatic extraction, the socket was allowed to heal for 3 months. At the delayed implant placement visit, adequate bone volume was confirmed radiographically, and a bone-level implant was placed with an insertion torque of 40 N·cm. At 1-year follow-up, the implant demonstrated successful osseointegration with PD of 3.2 mm, mPLI of 1.0, and mSBI of 0.5. The patient reported a total OHIP-14 score of 4.2 and VAS satisfaction scores averaging 8.1 across all domains. Radiographic evaluation showed stable marginal bone levels with minimal resorption.

#### Case 2 (observation group – immediate implantation)

A 39-year-old male patient presented with a fractured maxillary central incisor following trauma. Immediate implant placement was performed at the time of extraction with careful socket preparation and primary stability was achieved at 38 N·cm insertion torque. The jumping gap was grafted with deproteinised bovine bone mineral and covered with a resorbable membrane. At 1-year follow-up, excellent aesthetic and functional outcomes were achieved with PD of 2.9 mm, mPLI of 0.5, and mSBI of 0.5. The PES total score was 13, and the WES total score was 8, reflecting superior soft tissue contour and restoration aesthetics. The patient reported a total OHIP-14 score of 2.8 and VAS satisfaction scores averaging 9.2, particularly noting satisfaction with the reduced treatment time and maintained facial aesthetics throughout healing.

## DISCUSSION

This randomised controlled trial demonstrates that within 1 year, immediate and delayed implants achieve similar survival rates, while immediate implantation yields significantly better peri-implant health indices and patient-reported outcomes. The findings of this study support the null hypothesis regarding 1-year survival but reveal important differences in secondary outcomes that have substantial clinical relevance for patient care and treatment planning.

Dental implants have become a reliable treatment for replacing missing teeth with aesthetic and functional benefits and have a favourable long-term prognosis.^7,32^ Historically, delayed implantation was the most common surgical approach for patients with missing teeth treated with implant restorations, which was mainly performed after the patient had undergone extraction with a healing period of more than 3 months to allow for complete socket healing.^3,27^ In contrast, immediate implantation is an evolving technique in which the implant is placed immediately after tooth extraction, thus effectively shortening the duration of implant restoration and potentially reducing the occurrence of alveolar bone atrophy or resorption.^8,42^

In terms of implant survival rate, this study showed that the implant survival rates of the immediate implant group and the delayed implant group after 1 year were 95.45% and 92.73%, respectively, and the difference was not statistically significant (P = 0.391). This result aligns with previous systematic reviews and meta-analyses that concluded no significant difference in short-term implant survival rates between the two treatment modalities.^19,29^ Similarly, recent evidence comparing early versus delayed loading protocols has confirmed comparable survival outcomes.^49^ From the results of the present study, potential differences in alveolar bone remodelling patterns between immediate and delayed approaches did not significantly affect the 1-year implant survival rate. However, it should be emphasised that this study was only followed up for 1 year, and further extension of the follow-up period is necessary to observe the long-term implant survival rate and to evaluate whether the differences in bone remodelling patterns observed in the early period translate into clinically meaningful differences in long-term outcomes. The comparable short-term survival rates provide reassurance that immediate placement is a safe alternative when appropriate case selection criteria are met.

In this study, we found that 1 year after implantation, the levels of PD, mPLI, and mSBI were significantly lower than those before implantation in both groups, and importantly, the observation group demonstrated significantly greater improvements compared to the control group. This indicates that immediate implantation has measurable advantages in improving periodontal conditions. Several mechanisms may explain these findings. Immediate implantation reduces the exposure time of the extraction socket by placing the implant at the time of extraction, which can reduce the total number of surgical interventions, shorten the overall treatment time, reduce cumulative bacterial exposure, and facilitate the healthy recovery of periodontal tissues.^12,50^ Additionally, immediate implantation reduces the duration of physiologic bone remodelling after extraction and maintains a better alveolar bone morphology and dimension, which may improve the stability and health of peri-implant tissues.^31^ These findings are consistent with recent research demonstrating that preservation of socket dimensions through immediate placement can lead to more favourable peri-implant tissue architecture. The superior periodontal outcomes observed in this study align with recommendations from the Implant Dentistry Core Outcome Set, emphasising the importance of soft tissue health as a key parameter in evaluating implant success.^45^

In this study, PES and WES scores were used to assess the aesthetic outcome according to standardised protocols, and the results showed that 1 year after implantation, the total PES and WES scores of patients in both groups were significantly higher than those before implantation, with immediate implantation demonstrating significantly enhanced aesthetic outcomes compared to delayed placement. The advantages of immediate implantation in terms of aesthetic effect may stem from its ability to restore the appearance and function of teeth soon after extraction, minimising the impact of missing teeth on the patient’s facial aesthetics during the healing period.^11,15,29^ At the same time, immediate implantation better preserves the soft and hard tissues around the extraction socket, maintaining the original tissue architecture and providing a more favourable foundation for aesthetic reconstruction of the soft tissues around the implant.^2,21,33^ This is particularly important in the aesthetic zone, where tissue preservation is critical for optimal outcomes. In contrast, delayed implants necessitate a waiting period during which the extraction site undergoes physiological remodelling of the hard and soft tissues, which can lead to dimensional changes of the alveolar ridge in both height and width, potentially affecting the final aesthetic result and requiring additional grafting procedures.^4^ Recent systematic reviews on post-extraction ridge preservation support the concept that immediate intervention can minimise tissue loss.^1^ The superior aesthetic scores observed in the immediate group in this study are consistent with findings from other investigations demonstrating the benefits of immediate placement for maintaining soft tissue contours and papilla fill.

Functional limitations have been reported to be most affected by missing teeth, followed by psychological discomfort and physical pain.^6^ It was found that a poorer quality of life assessed before treatment in patients with missing teeth was most strongly associated with psychological discomfort. The main reason for this may be that missing teeth can impose a burden on the psychological and financial aspects of the patient, as well as affecting facial contour and appearance, especially for anterior teeth, which creates certain barriers to socialisation and causes the patient to feel self-conscious in social situations, resulting in psychological discomfort and reduced confidence. In addition, there can be significant discomfort after tooth extraction and implant surgery, causing patients to have difficulty eating and affecting their daily activities.^26,36^ The results of this study showed that all OHIP-14 scores and total scores of both groups were significantly lower (improved) compared to baseline before implantation, and the functional limitations, physical pain, psychological discomfort, psychological disorders, and total OHIP-14 scores of the observation group were significantly lower than those of the control group. This indicates that both implant modalities can significantly improve the quality of life of patients, but immediate implantation is significantly more effective in improving multiple dimensions of quality of life. Immediate implantation substantially shortens the duration of the edentulous period, enabling patients to recover chewing function and aesthetics more rapidly, and reduces functional limitations and physical discomfort caused by the period of missing teeth.^33^ At the same time, immediate implantation also provides patients with a positive psychological impact, alleviating the psychological discomfort and social barriers caused by tooth loss, thus improving the overall quality of life of patients.^14,18^ These quality of life improvements are particularly meaningful from a patient-centred care perspective. In addition, VAS was used to assess patient satisfaction across multiple domains, and the results showed that immediate implant patients were able to restore normal oral function and aesthetics more quickly with fewer treatment visits, reducing discomfort and inconvenience during the treatment process, and thus were significantly more satisfied with the treatment outcomes.^15,16^ The patient-reported outcome measures used in this study align with recommendations from recent consensus statements emphasising the importance of incorporating patient perspectives in evaluating implant treatment success.^45^ Higher patient satisfaction in the immediate group reflects not only the functional and aesthetic outcomes but also the psychological benefits of reducing treatment time and avoiding the social impact of extended edentulism.

These findings have important clinical implications for treatment planning. Clinicians should consider immediate implant placement as a preferred option when adequate primary stability can be achieved, particularly in patients who are concerned about treatment duration, aesthetics during healing, and maintaining social function. Long-term success of implant therapy requires systematic monitoring using validated diagnostic measures and standardised radiographic assessment protocols to ensure optimal peri-implant health and early detection of potential complications.^10,34^ The comparable survival rates combined with superior secondary outcomes suggest that immediate placement offers a favourable benefit-to-risk ratio in appropriately selected cases. Patient counselling should emphasise the potential advantages of immediate placement, including reduced treatment time, better preservation of alveolar bone dimensions, improved aesthetic outcomes, and enhanced quality of life, while acknowledging that both approaches are effective. For cases where immediate placement is technically feasible and adequate primary stability can be obtained, the evidence from this study supports its use. Conversely, delayed placement remains an appropriate and successful approach when anatomical or clinical conditions do not favour immediate placement. Future treatment algorithms may incorporate patient preferences for treatment duration and aesthetic concerns during healing as important factors in the decision-making process.

This study also has several important limitations that should be acknowledged. First, although the sample size was adequate based on power calculation for the primary outcome, additional studies with larger cohorts across multiple centres would enhance the generalizability of findings. Second, and more critically, the follow-up period of this study was only 1 year. Further extension of the follow-up period is essential to study the long-term implant survival rate, assess the long-term stability of the periodontal condition, evaluate the long-term maintenance of the aesthetic effect, and determine whether the observed advantages of immediate placement persist over time or whether delayed placement outcomes converge with extended follow-up. Long-term data extending to 5 years and beyond would provide valuable information about the durability of these differences. Third, because the OHIP-14 short-form may not fully capture domain-specific impacts with the same sensitivity as the original 49-item version, interpretation of subscale differences should be undertaken with appropriate caution. Fourth, our single-centre cohort from a specialised dentistry setting may limit extrapolation of OHIP-14 performance and treatment outcomes to non-dental populations or different clinical settings within the broader Chinese population. Geographic and socioeconomic diversity should be represented in future studies. Fifth, the OHIP-14 is a self-reported measure that may be influenced by recall bias and social desirability factors, though the validated Chinese version used in this study has demonstrated strong psychometric properties. Sixth, although multivariate adjustment was applied for key demographic and clinical variables, residual confounding from unmeasured socioeconomic, psychological, or behavioural factors cannot be fully excluded. Seventh, the study included only single-tooth replacements, and findings may not be directly applicable to multiple adjacent tooth replacements or more complex clinical scenarios. Eighth, operator experience and individual surgical technique variations, while standardised in this study, may influence outcomes in broader clinical practice. Finally, both treatment protocols used in this study followed standardised approaches; however, there are multiple variations of immediate and delayed placement protocols described in the literature, and the specific surgical techniques employed may influence comparative outcomes.

In conclusion, this randomised controlled trial demonstrates that there is no significant difference between immediate and delayed implantation in the 1-year success rate of dental implant patients, confirming comparable short-term survival outcomes. However, immediate implantation offers significant clinical advantages in improving periodontal-related indexes, aesthetic effects, quality of life and patient satisfaction. These findings suggest that when adequate primary stability can be achieved and appropriate case selection criteria are met, immediate implantation represents a favourable treatment option that provides patients with enhanced clinical and patient-reported outcomes while maintaining equivalent survival rates. Clinicians should consider these multiple dimensions of treatment success when counselling patients and developing individualised treatment plans. Future studies should expand the sample size across multiple centres, extend the follow-up period to at least 5 years to evaluate long-term stability and survival, and explore the application effects and comparative outcomes of immediate implantation and delayed implantation in patients with different types of tooth loss, varying anatomical conditions, and diverse patient populations, thereby providing an increasingly comprehensive and reliable evidence base for oral implantation clinical practice and enhancing patient-centred treatment decision-making.

### Acknowledgements

#### Funding

No funding was received.

#### Availability of data and materials

The data sets used and/or analysed during the current study are available from the corresponding author on reasonable request.

#### Ethics approval

The study was approved by the Ethics Committee of General Hospital of Central Theater Command (GN-CTC-121).

#### Conflict of interest

The authors declare that they have no conflict of interest.
